# Commentary: Different immunological mechanisms govern protection from experimental stroke in young and older mice with recombinant TCR ligand therapy

**DOI:** 10.3389/fncel.2014.00339

**Published:** 2014-10-17

**Authors:** Keith R. Pennypacker

**Affiliations:** Molecular Pharmacology and Physiology, University of South FloridaTampa, FL, USA

**Keywords:** experimental stroke, aging, RTL1000, therapy, immune response, neuroinflammation

Translational research to discover therapeutic targets for stroke has not fared well in developing new treatments for patients. The three most pressing issues are the choice of therapeutic target, animal model, and timepoint of administration of treatment. This manuscript “Different immunological mechanisms govern protection from experimental stroke in young and older mice with recombinant TCR ligand therapy” provides a fresh approach toward developing stroke treatments (Offner et al., 2014). This study examines treating with recombinant TCR ligand (RTL) in both young and older mice 4 hours after MCAO. RTL consist of the specific domains of MHC II molecule and inhibits activation of T cells toward inflammation. The novelties of this study are several fold including: (1) inhibition of a specific immunoinflammatory pathway to avoid total immunosuppression, (2) use of elderly mice to mimic the population of human patients and (3) administration at a clinically relevant timepoint.

For a number of years, researchers have reported the entry of immune cells into the area of the infarct, which leads to further neurodegeneration. However, targeting this immune response as a treatment for stroke remains elusive (Iadecola and Anrather, 2011). In the past several years, the spleen has been reported to be a focal point for the immune system to mount an inflammatory response that exacerbates stroke-induced neurodegeneration (Offner et al., 2006; Vendrame et al., 2006). In fact, ablation of the spleen reduces inflammation and neural cell death in the rodent brain after experimental stroke (Ajmo et al., 2008; Ostrowski et al., 2012; Jin et al., 2013). Blockade of interferon gamma signaling has been reported to be neuroprotective in experimental stroke (Liesz et al., 2009, 2011; Seifert et al., 2014) although other groups have reported alternative results in their model systems (Chu et al., 2000; Lambertsen et al., 2004). T cells are the main effector cell of this neurodegenerative response and responsible for the release of the proinflammatory interferon gamma, which is a potent activator of microglia becoming neurotoxic (Boehm et al., 1997; Mebius and Kraal, 2005). Moreover, addition of interferon gamma reverses the neuroprotection provided by splenectomy, demonstrating that this cytokine plays a major role in the spleen eliciting response resulting in further neural death after stroke (Seifert et al., 2012). As shown in this study, administration of RTL directly inhibits the T cell response to stroke, which concomitantly deactivates the splenic response as well in both young and old mice.

This treatment reduced infarct volumes in both age groups by altering the immune response to this neurological insult. Most interestingly, the recombinant TCR ligand differentially affected components of the immune response in the elderly and young mice but still resulted in a blunted immune response reducing neurodegeneration. This study illuminated differences in the immune cell composition and inflammatory expression between these two age groups in response to stroke. This is one of the few studies that enlists elderly animals which begins to provide insight into the differences in the physiological responses to stroke between young and the aged animals. Such differences in young and elderly could be responsible for the failure to translate findings at the bench to those in the clinic since the vast majority of studies use greatly cheaper young ones. More studies using elderly rodents will further supply additional insight in translating stroke treatment from the preclinical research to the clinical setting. This study sets the basis for future ones to develop new therapeutic approaches for a treatment for stroke.

## Conflict of interest statement

The author declares that the research was conducted in the absence of any commercial or financial relationships that could be construed as a potential conflict of interest.

## References

[B1] Ajmo C. T. Jr. Vernon D. O. Collier L. Hall A. A. Garbuzova-Davis S. Willing A.. (2008). The spleen contributes to stroke-induced neurodegeneration. J. Neurosci. Res. 86, 2227–2234. 10.1002/jnr.21661 18381759 PMC2680137

[B2] Boehm U. Klamp T. Groot M. Howard J. C. (1997). Cellular responses to interferon-gamma. Annu. Rev. Immunol. 15, 749–795. 10.1146/annurev.immunol.15.1.749 9143706

[B3] Chu C. Q. Wittmer S. Dalton D. K. (2000). Failure to suppress the expansion of the activated CD4 T cell population in interferon gamma-deficient mice leads to exacerbation of experimental autoimmune encephalomyelitis. J. Exp. Med. 192, 123–128. 10.1084/jem.192.1.123 10880533 PMC1887710

[B4] Iadecola C. Anrather J. (2011). The immunology of stroke: from mechanisms to translation. Nat. Med. 17, 796–808. 10.1038/nm.2399 21738161 PMC3137275

[B5] Jin R. Zhu X. Liu L. Nanda A. Granger D. N. Li G. (2013). Simvastatin attenuates stroke-induced splenic atrophy and lung susceptibility to spontaneous bacterial infection in mice. Stroke 44, 1135–1143. 10.1161/STROKEAHA.111.000633 23391769 PMC3609888

[B6] Lambertsen K. L. Gregersen R. Meldgaard M. Clausen B. H. Heibol E. K. Ladeby R.. (2004). A role for interferon-gamma in focal cerebral ischemia in mice. J. Neuropathol. Exp. Neurol. 63, 942–955. 15453093 10.1093/jnen/63.9.942

[B7] Liesz A. Suri-Payer E. Veltkamp C. Doerr H. Sommer C. Rivest S.. (2009). Regulatory T cells are key cerebroprotective immunomodulators in acute experimental stroke. Nat. Med. 15, 192–199. 10.1038/nm.1927 19169263

[B8] Liesz A. Zhou W. Mracsko E. Karcher S. Bauer H. Schwarting S.. (2011). Inhibition of lymphocyte trafficking shields the brain against deleterious neuroinflammation after stroke. Brain 134(Pt 3), 704–720. 10.1093/brain/awr008 21354973

[B9] Mebius R. E. Kraal G. (2005). Structure and function of the spleen. Nat. Rev. Immunol. 5, 606–616. 10.1038/nri1669 16056254

[B10] Offner H. Dotson A. L. Zhu W. Libal N. Alkayed N. J. (2014). Different immunological mechanisms govern protection from experimental stroke in young and older mice with recombinant TCR ligand therapy. Front. Cell. Neurosci. 8:284. 10.3389/fncel.2014.00284 PMC4174768 25309326

[B11] Offner H. Subramanian S. Parker S. M. Wang C. Afentoulis M. E. Lewis A.. (2006). Splenic atrophy in experimental stroke is accompanied by increased regulatory T cells and circulating macrophages. J. Immunol. 176, 6523–65231. 10.4049/jimmunol.176.11.6523 16709809

[B12] Ostrowski R. Schulte R. Nie Y. Ling T. Lee T. Manaenko A.. (2012). Acute splenic irradiation reduces brain injury in the rat focal ischemic stroke model. Transl. Stroke Res. 3, 473–481. 10.1007/s12975-012-0206-5 23956805 PMC3743446

[B13] Seifert H. A. Collier L. A. Chapman C. B. Benkovic S. A. Willing A. E. Pennypacker K. R. (2014). Pro-inflammatory interfon gamma signaling is directly associated with stroke induced neurodegeneration. J. Neuroimmune Pharmacol. [Epub ahead of print]. 10.1007/s11481-014-9560-2 25104571 PMC4209188

[B14] Seifert H. A. Leonardo C. C. Hall A. A. Rowe D. D. Collier L. A. Benkovic S. A.. (2012). The spleen contributes to stroke induced neurodegeneration through interferon gamma signaling. Metab. Brain Dis. 27, 131–141. 10.1007/s11011-012-9283-0 22354752 PMC4739736

[B15] Vendrame M. Gemma C. Pennypacker K. R. Bickford P. C. Davis Sanberg C. Sanberg P. R.. (2006). Cord blood rescues stroke-induced changes in splenocyte phenotype and function. Exp. Neurol. 199, 191–200. 10.1016/j.expneurol.2006.03.017 16713598

